# Sequencing Biodegradable and Potentially Biobased Polyesteramide of Sebacic Acid and 3-Amino-1-propanol by MALDI TOF-TOF Tandem Mass Spectrometry

**DOI:** 10.3390/polym14081500

**Published:** 2022-04-07

**Authors:** Paola Rizzarelli, Stefania La Carta, Emanuele Francesco Mirabella, Marco Rapisarda, Giuseppe Impallomeni

**Affiliations:** 1 Istituto per i Polimeri, Compositi e Biomateriali, Consiglio Nazionale delle Ricerche, Via Paolo Gaifami 18, 95126 Catania, Italy; emanuelefrancesco.mirabella@cnr.it (E.F.M.); marco.rapisarda@ipcb.cnr.it (M.R.); giuseppe.impallomeni@cnr.it (G.I.); 2 STMicroelectronics Srl, Stradale Primosole, 50, 95121 Catania, Italy; stefanialacarta@gmail.com

**Keywords:** polyesteramides, biodegradable polymers, biobased polymers, mass spectrometry, MALDI, tandem mass spectrometry, sequencing, characterization, Py-GC/MS

## Abstract

Biodegradable and potentially biobased polyesteramide oligomers (PEA-Pro), obtained from melt condensation of sebacic acid and 3-amino-1-propanol, were characterized by nuclear magnetic resonance (NMR), matrix assisted laser desorption/ionization-time of flight/time of flight-mass spectrometry/mass spectrometry (MALDI-TOF/TOF-MS/MS), thermogravimetric analysis (TGA), and pyrolysis-gas chromatography/mass spectrometry (Py-GC/MS). NMR analysis showed the presence of hydroxyl and amino terminal groups as well as carboxylic groups of the sebacate moiety. Hydroxyl and carboxyl termination had the same abundance, while the amine termination was 2.7-times less frequent. Information regarding the fragmentation pathways and ester/amide bond sequences was obtained by MALDI-TOF/TOF-MS/MS analysis performed on sodiated adducts of cyclic species and linear oligomers. Different end groups did not influence the observed fragmentation. Three fragmentation pathways were recognized. The β-hydrogen-transfer rearrangement, which leads to the selective scission of the –O–CH_2_– bonds, was the main mechanism. Abundant product ions originating from –CH_2_–CH_2_– (β–γ) bond cleavage in the sebacate moiety and less abundant ions formed by –O–CO– cleavages were also detected. TGA showed a major weight loss (74%) at 381 °C and a second degradation step (22% weight loss) at 447 °C. Py-GC/MS performed in the temperature range of 350–400 °C displayed partial similarity between the degradation products and the main fragments detected in the MALDI-TOF/TOF-MS/MS experiments. Degradation products derived from amide bonds were related to the formation of CN groups, in agreement with the literature.

## 1. Introduction

Aliphatic polyesteramides represent a class of biodegradable and potentially biobased polymers. They combine the mechanical and thermal properties of polyamides with the high biodegradability of polyesters, leading to materials with better properties and processability compared to polyesters with similar structures [1].

The detailed characterization of synthetic polymers plays an important role in defining the chemical structure and its influence on material properties, as well as its utilization. During the last few years, matrix-assisted laser desorption/ionization time-of-flight mass spectrometry (MALDI-TOF MS) has become a routine analytical technique for the analysis of synthetic polymers, providing more and more detailed structural information with high sensitivity, thanks to improved technologies and analytical strategies [2,3]. It has been successfully used in both characterization and degradation studies, with some limitations in the accuracy of the measured molecular weight related to the polydisperse nature of polymers, especially when dealing with high mass range [4]. Despite MALDI being a soft ionization technique, it is possible to obtain fragment ion spectra through the so-called post-source decay (PSD) [5]. MALDI-PSD has been successfully applied for peptide sequencing and biopolymers analysis, but to a lesser extent for synthetic polymers, due to their greater complexity. The development of the MALDI-TOF/TOF (Matrix-assisted laser desorption ionization time-of-flight/time-of-flight) mass spectrometer, with the aim of simplifying and improving the experimental procedures and acquiring more reliable information, bypassed many limits of the PSD and collision-induced dissociation (CID) in a conventional MALDI instrument. In a MALDI-TOF/TOF mass spectrometer operating in tandem mass spectrometry mode (MALDI-TOF/TOF-MS/MS), the high-speed ions separated in the first TOF analyser are selected by a timed ion gate, collided with atoms or molecules, and accelerated for analysis in the second TOF mass analyser fitted with a two-stage reflector [6].

MS/MS is increasingly applied to analyse synthetic polymers since it can provide information on chain-end or in-chain substituents, discriminate isobaric and isomeric species, differentiate linear and cyclic polymers, and establish macromolecular connectivity, sequences and architectures [7,8]. In fact, in several cases, single-stage mass data may not be sufficient to unequivocally establishing the polymer structure. For confident structural assignments, fragmentation studies provide initial information to help understand tandem mass spectra. In fact, knowledge of the fragmentation mechanisms of polymer ions provides guidelines on how to obtain the desired information from the product ions detected in MS/MS spectra and to deduce the real macromolecular architecture [9]. Amongst the MS/MS tools, the collision-induced dissociation (CID) approach represents the preferred technique for the deep structural characterization of gas-phase ions.

MALDI-TOF/TOF-MS/MS has been successfully applied to the analysis of biodegradable, as well as polyesters and polyesteramides [10,11]. It provided structural information regarding the sequence of ester and amide bonds in synthetic polyesteramides [12,13] and the fragmentation pathways in biodegradable polyesters [14,15].

In this work, Nuclear magnetic resonance (NMR) analysis showed the nature of the terminal groups and allowed their quantification. MALDI-TOF MS revealed the presence of cyclic species and linear oligomers with the main end groups and additional ones which eluded NMR analysis. MALDI-TOF/TOF-MS/MS was employed to investigate the ester/amide bond sequences and the fragmentation mechanisms of a polyesteramide (PEA-Pro) from the melt condensation of 3-amino-1-propanol and sebacic acid that can be produced from a renewable source (castor oil). The MALDI-TOF/TOF-MS/MS analysis was performed on cyclic species and linear oligomers with different end groups, selecting as precursor ions the sodium adducts of the oligomers identified in the MALDI-TOF/TOF spectra.

Furthermore, pyrolysis-gas chromatography/MS (Py-GC/MS) was applied to confirm the results acquired by MALDI-TOF/TOF. Pyrolysis technique hyphenated to GC/MS is the method most frequently used for polymer characterization and for studying their thermal degradation processes [16]. Py-GC/MS provides valuable information about the molecular structure, and it is a particularly useful method for the analysis of complex blends of traditional and biodegradable macromolecules [17,18]. Moreover, we used thermogravimetric analysis (TGA) to check the degradation steps and establish the best experimental conditions in Py-GC/MS analyses.

## 2. Materials and Methods

### 2.1. Materials

Low molecular weight polyesteramide (PEA-Pro) was synthesized by melt polymerization starting from sebacic acid and 3-amino-1-propanol, using stoichiometric amounts of reagents as described in [19]. 2-(4-Hydroxyphenylazo)benzoic acid (HABA), reagents, and solvents were obtained from Sigma-Aldrich Chemical Co. (Milan, Italy) and used as received.

### 2.2. Nuclear Magnetic Resonance (NMR)

NMR analysis was conducted with a ^UNITY^INOVA Agilent spectrometer (Santa Clara, CA, USA) operating at 500 MHz (^1^H), using standard Agilent pulse sequences. Spectra were elaborated with the MestreNova software (Mestrelab Research, Santiago de Compostela, Spain). The sample was dissolved in deuterated dimethylsulfoxide (6d-DMSO) and spectra acquired at 50 °C and calibrated with the solvent residual ^1^H signal set at 2500 ppm.

### 2.3. MALDI Sample Preparation

The polyesteramide sample was dissolved in 1,1,1,3,3,3-hexafluoro-2-propanol (HFIP) at a concentration of di 3 mg/mL. HABA (0.1 M in HFIP) was used as the matrix. Appropriate volumes of polymer solution and matrix solution were mixed to obtain 1:1, 1:2, 1:3 ratios (sample/matrix, *v*/*v*). An aliquot of 1 µL of each sample/matrix mixture was spotted onto the MALDI sample holder and slowly dried to allow matrix crystallization.

### 2.4. MALDI-TOF/MS Analysis

An Applied Biosystems 4800 MALDI TOF/TOF^TM^ Analyser (Framingham, MA, USA) mass spectrometer was employed in this study to acquire MALDI and MS/MS spectra with and without using a collision gas (air or argon). This TOF/TOF instrument is equipped with a Nd:YAG laser with 355 nm wavelength, pulse of <500 ps, and 200 Hz repetition rate in both MS and MS/MS modes. The precursor ion selector of the analyser has a mass resolution of about 400. All measurements were performed in automatic mode. For MS/MS experiments, the potential difference between the source acceleration voltage and the collision cell was set at 2 kV. MALDI-MS e MALDI-TOF/TOF-MS/MS spectra were recorded in reflector positive ion mode. MS and MS/MS data were processed using Data Explorer 4.4 (Applied Biosystems, Framingham, MA, USA). Mass resolution (FWMH) was about 13,000 in MS mode and ranged from 1500 (*m*/*z* < 500) to 5000 (500 < *m*/*z* < 1300) in MS/MS mode.

### 2.5. Thermogravimetric Analysis (TGA)

TGA was performed with a TGA Q500 (TA Instruments, New Castle, DE, USA), using a platinum pan. Dynamic measurement was performed from 50 to 800 °C at 10 °C/min, under a nitrogen atmosphere (flow rate 60 mL/min). Sample weight was approximately 5 mg. The weight loss percent and its derivate (DTG) was recorded as a function of temperature.

### 2.6. Pyrolysis-Gas Chromatography/Mass Spectrometry Analysis (Py-GC/MS)

Py-GC/MS was performed on a small amount of sample (about 0.1 mg). It was placed, without pre-treatment, in the crucible immediately prior to analysis. Py-GC/MS was performed using a Multi-Shot Pyrolizer (EGA/PY-303D, Frontier Labs Ltd., Fukushima, Japan), connected to a GC system GC-2020 (Shimadzu Italia S.r.l., Milan, Italy), coupled with to a triple quadrupole mass spectrometer detector and with electronic ionization (70 eV) Mass Detector TQ8040 (Shimadzu Italia S.r.l., Milan, Italy).

The gas chromatography was equipped with Ultra Alloy^®^ Metal Capillary Column (Frontier Labs, stationary phase 5% di-phenyl-methylpolysiloxane, with an inner diameter of 250 μm, a film thickness of 0.25 μm and a length of 30 m). Interfaces of Py-GC and GC/MS were kept at 300 °C and 250 °C, respectively. Several experiments were performed setting different combination steps of pyrolysis between 350 and 450 °C. Oven temperature was held at 50 °C for 1 min, increased to 100 °C at 30 °C/min, then held at 100 °C for 5 min, ramped from 100 °C to 300 °C at 10 °C/min and finally it was maintained at 300 °C for 10 min. The carrier gas was helium at a controlled flow of 1.78 mL/min. The split ratio was 1/50 of the total flux. Mass range was set from *m*/*z* 35 up to 500. Blanks were carried by placing the crucible empty in the furnace and performing pyrolysis in the conditions mentioned above. Structure assignment was carried out running the similarity search routine of the instrument.

## 3. Results

### 3.1. NMR Analysis

PEA-Pro was investigated by NMR in order to confirm its structure and the nature and abundance of terminal groups.

**Figure 1:**
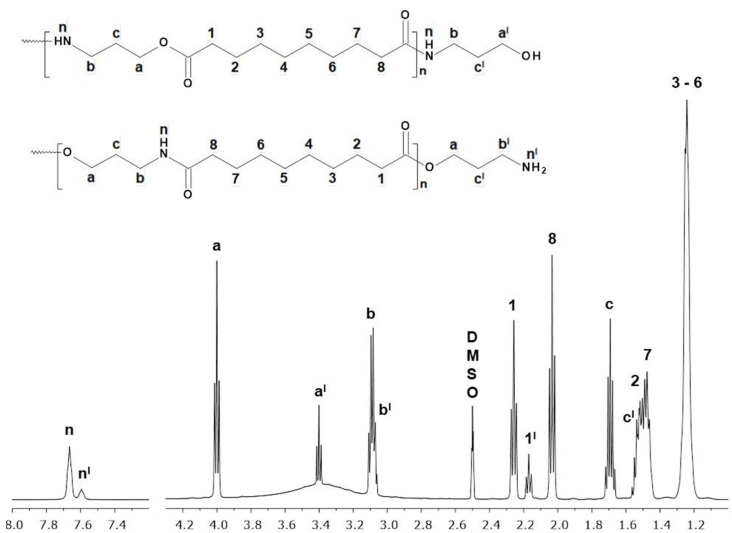
^1^H-NMR spectrum of PEA-Pro in 6d-DMSO at 50 °C. Signal 1′ is assigned to the sebacate methylene protons next to a terminal carboxylic group.

The ^1^H-NMR spectrum of PEA-Pro in 6d-DMSO, together with its chemical assignments, is shown in Figure 1. A gradient-enhanced COSY spectrum (see Supplementary Material Figures S1 and S2) helped with signal interpretation. The 3-amino-1-propanol resonances in the polymer chain were found at 7.665, 4.000, 3.089, and 1.692 ppm for protons (n), (a), (b), and (c), and were accompanied by lower signals at 7.595, 3.401, 3.079, and 1.536 ppm assigned to the same protons when the amino-alcohol was in terminal position (signals n’, a’, b’, and c’). The sebacate unit gave resonances at 2.258 ppm due to the methylene next to the ester group (protons 1 in Figure 1), at 2.033 ppm due to the methylene next to the amide group (protons 8 in Figure 1), at 1.512 and 1.476 ppm due to methylene 2 and 7. The large singlet at 1.243 ppm was assigned to the four central methylene groups, from 3 to 6, and the small triplet at 2.170 ppm to the methylene next to the carboxylic group of a terminal sebacate unit (signal 1′ in Figure 1). Finally, a visible hump of the baseline was present at about 3.4 ppm, which can be assigned to hydroxyl groups of terminal 3-amino-1-propanol.

These results showed that the PEA-Pro structure is consistent with its chemical synthesis and that it has carboxylic and 3-amino-1-propanol termination. The latter may present a free hydroxyl or amine group and, from a quantitative point of view, hydroxyl and carboxyl termination have the same abundance, while the amine termination is 2.7-times less frequent.

### 3.2. MALDI-TOF/TOF Analysis

**Figure 2:**
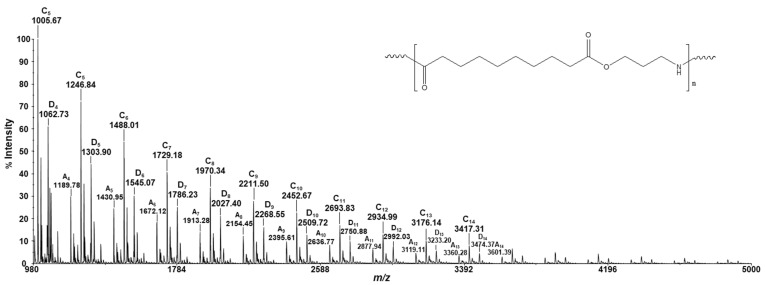
MALDI/TOF-TOF mass spectrum in reflector mode of the PEA-Pro sample.

The MALDI-TOF/TOF mass spectrum of PEA-Pro, recorded in reflector mode, is shown in Figure 2. The spectrum, which extends up to *m*/*z* 5000, consists of a distribution of ions corresponding to singularly-charged sodiated and potassiated adducts. The most abundant ion series is due to linear PEA-Pro chains terminated with carboxyl and amino-alcohol end groups (species C), followed by linear chains terminated by amino-alcohol (species D) or sebacic acid (species A) at both ends. The repeat unit of PEA-Pro is shown in the same picture and its mass (241.17 Da) matches the difference between the *m*/*z* values of species belonging to consecutive clusters. The repeat unit reported in Figure 2 is not necessarily representative of the real bond sequences in the polyesteramide chains. It was arbitrarily chosen to report the ester/amide bond sequence as alternating; during the synthesis, however, ester/amide, ester/ester and amide/amide units can be produced in a random sequence, as already mentioned for other polyesteramides [12,13].

**Figure 3:**
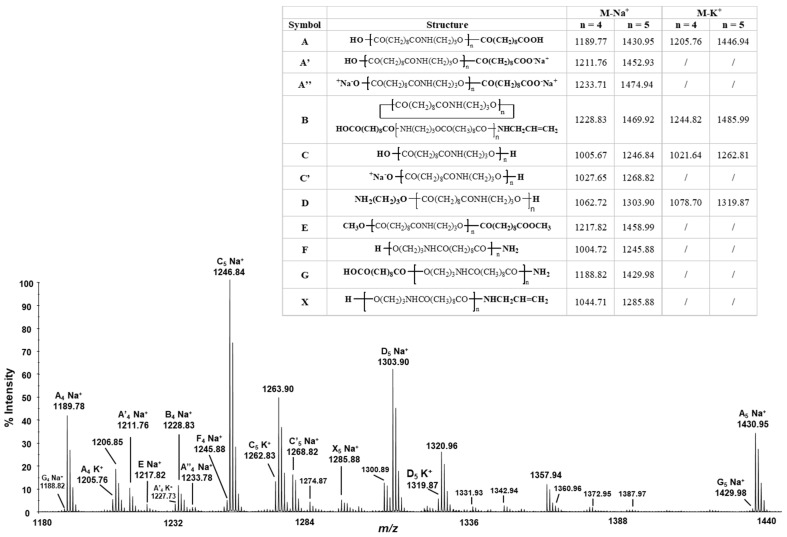
Enlarged portion of the MALDI/TOF-TOF mass spectrum in reflector mode of the PEA-Pro sample.

An enlarged portion of this mass spectrum, in the *m*/*z* range 1180–1440, is reported in Figure 3, together with a table showing the structures of the identified species. Despite dimethyl sebacate and 3-amino-1-propanol being employed as starting monomers, carboxylic end groups were identified; their presence is explained through the hydrolysis of terminal or inner ester groups. The chains terminated with amino-alcohol groups can bear a hydroxyl (–NH(CH_2_)_3_OH) or an amine (–O(CH_2_)_3_NH_2_) end group that cannot be discriminated by mass spectrometry. Sodiated species at *m*/*z* 1228.8 are due to linear chains bearing a carboxyl end group and an olefin end group (CH_2_=CHCH_2_NH–). These chain ends are formed by thermal degradation processes taking place during the synthesis of the polymer sample [16].

Fifteen MALDI ions in Figure 3 can be assigned unambiguously due to eight oligomers.

1. Sodiated and potassiated linear di-carboxyl terminated chains (species A Na^+^, *m*/*z* 1189.77–1430.95; species A K^+^, *m*/*z* 1205.76–1446.94) and the corresponding sodium salt (A’ Na^+^, *m*/*z* 1211.76–1452.93) and disodium salt of the same species (A’’ Na^+^, *m*/*z* 1233.71–1474.94);
2. Sodiated and potassiated cyclic PEA-Pro chains (species B Na^+^, *m*/*z* 1228.83; B K^+^, *m*/*z* 1244.82).
3. Sodiated and potassiated linear oligomers terminated with carboxyl at one end and amino-alcohol groups at the other end (species C Na^+^, *m*/*z* 1246.84; species C K^+^, *m*/*z* 1262.81), and the corresponding sodium salt of the same species (C’ Na+, *m*/*z* 1268.82);
4. Sodiated and potassiated linear di-amino-alcohol terminated oligomers (species D Na^+^ *m*/*z* 1303.90, species D K^+^, *m*/*z* 1319.87);
5. Sodiated linear oligomers terminated with dimethyl ester of sebacic acid at both the ends (species E Na^+^ *m*/*z* 1217.82);
6. Sodiated linear oligomers terminated with amino-alcohol at one end and –NH_2_ group at the other end (species F Na^+^ *m*/*z* 1245.88);
7. Sodiated linear oligomers terminated with sebacic acid at one end and –NH_2_ group at the other end (species G Na^+^ *m*/*z* 1429.98);
8. Sodiated linear oligomers terminated with amino alcohol at one end and olefin derived by amino alcohol at the other end (species X Na^+^ *m*/*z* 1285.88).

The structures of these eight oligomers, identified in the inset of Figure 3 are predictable according to the method of synthesis and thermal degradation mechanisms (species G and F) [20]. Moreover, ions at *m*/*z* 1360.96 are reasonably due to end groups bearing ether bonds, i.e., H[U]_4_NH(CH_2_)_3_O(CH_2_)_3_NH_2_. Likewise, ions at *m*/*z* 1342.94 can be assigned to cyclic species with two ether bonds in the macromolecular chain. Noticeably, oligomers of unknown structure are also observed (*m*/*z* 1263.90, 1274.87, 1300.89, 1320.96, 1331.93, 1357.94, 1372.95, 1387.97) and their presence could be related to additional side reactions taking place in the polymerization process.

### 3.3. MALDI-TOF/TOF-MS/MS Analysis

Four sodiated oligomers were selected as precursor ions to perform MALDI-TOF/TOF-MS/MS analysis: three linear oligomers terminated, respectively, by two carboxylic groups (*m*/*z* 1189.8, species A, Figure 3), a carboxyl and an amino-alcohol group (*m*/*z* 1246.8, species C, Figure 3), and two amino-alcohol groups (*m*/*z* 1303.9, species D, Figure 3) and, finally, a cycle (*m*/*z* 1228.8, species B, Figure 3).

All the MALDI-TOF/TOF-MS/MS spectra acquired show a similar (most abundant) series of product ions. Then, the fragmentation pathway is not affected by different end groups.

**Scheme 1:**
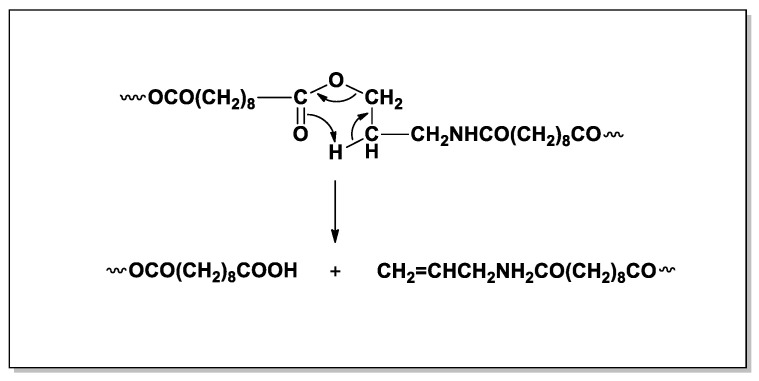
β-hydrogen transfer mechanism.

As in previous works [21], the β-hydrogen transfer rearrangement appears to be the main fragmentation mechanism (Scheme 1). It is a typical mechanism of thermal degradation in polyesters [16] and involves the transfer of a hydrogen in β position to the carboxyl group of the diacid via a six-membered cyclic transition state. As a consequence, two ions are obtained, one bearing a carboxylic acid end group and the other terminated with an olefin end group (CH_2_=CHCH_2_NHCO–). The sodium affinity of the species formed in the dissociation reaction can result in a different abundance of the product ions in the MALDI-TOF/TOF-MS/MS spectrum.

**Figure 4:**
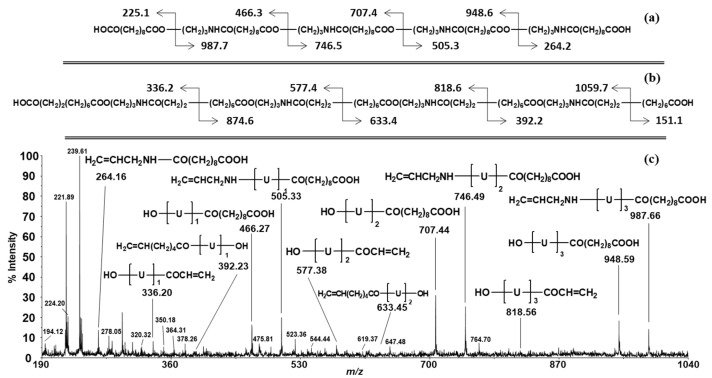
*m*/*z* values of the sodiated product ions originating from: (**a**) the *β*-hydrogen transfer and (**b**) the −CH_2_−CH_2_− (β–γ) bond cleavages in the sebacate moiety for the sodiated precursor ions at *m*/*z* 1189.8. (**c**) MALDI-TOF/TOF-MS/MS spectrum of the sodiated dicarboxyl-terminated oligomers at *m*/*z* 1189.8 of the PEA-Pro sample (U = −NH(CH_2_)_3_OCO(CH_2_)_8_CO− or −O(CH_2_)_3_NHCO(CH_2_)_8_CO−; collision gas = argon).

Figure 4 displays the MALDI-TOF/TOF-MS/MS spectrum of the sodiated di-carboxyl-terminated oligomers at *m*/*z* 1189.8 of the PEA-Pro sample together with the *m*/*z* values of the two main fragmentation pathways. The main product ions at *m*/*z* 225.10, 466.27, 707.44 and 948.59 and their complementary ions at *m*/*z* 264.16, 505.33 746.49 and 987.66 are due to *β*-hydrogen-transfer rearrangement, Figure 4a, typical for polyesters in thermal degradation. The less abundant product ions at *m*/*z* 336.20, 577.38, and 818.56 and their complementary 633.45, 392.23 and 151.10 arise from –CH_2_–CH_2_– (β–γ) bond cleavage in the sebacate moiety, Figure 4b. This scission is supported by the formation of product ions bearing a *α*,β-unsaturated ester or amide end group (CH_2_=CHCOO– or CH_2_=CHCONH–). Furthermore, unsaturated and saturated fragments should both be formed, but the saturated fragments are significantly less abundant. In Figure 4 only the *m*/*z* values of the unsaturated fragments are reported; the *m*/*z* values of the complementary saturated ions can be easily obtained. The mass spectrum in Figure 4 also displays clusters of ions at an interval of 14 (CH_2_) *m*/*z* units (e.g., *m*/*z* 336.17, 350.14, 364.41, 378.37, 392.41) that are typical of hydrocarbons and long-chain acids. All assigned structures are shown in Table S1.

**Figure 5:**
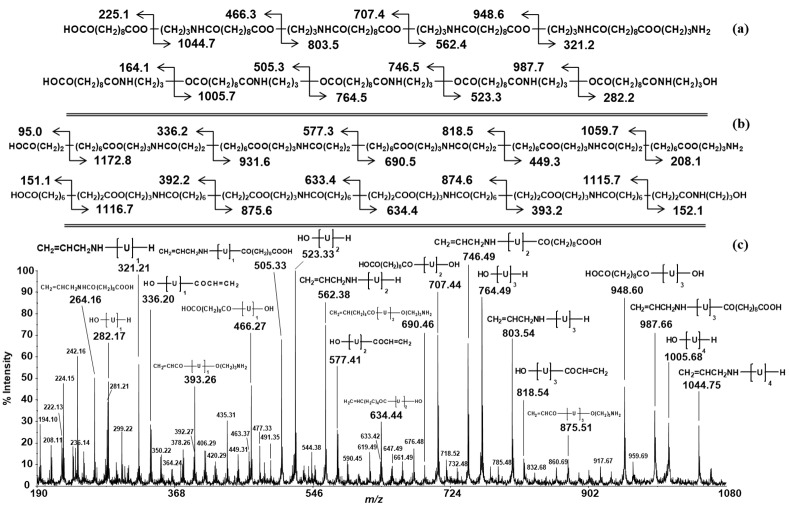
*m*/*z* values of the sodiated product ions originating from: (**a**) the *β*-hydrogen transfer and (**b**) the −CH_2_−CH_2_− (β−γ) bond cleavages in the sebacate moiety for the sodiated precursor ions at *m*/*z* 1246.8. (**c**) MALDI-TOF/TOF-MS/MS spectrum of the sodiated carboxyl and amino-alcohol terminated oligomers at *m*/*z* 1246.8 of the PEA-Pro sample (U= −NH(CH_2_)_3_OCO(CH_2_)_8_CO− or −O(CH_2_)_3_NHCO(CH_2_)_8_CO−; collision gas = argon).

Similar results were obtained from the MALDI-TOF/TOF-MS/MS analysis of the other precursor ions analysed. The mass spectrum of the sodiated carboxyl and amino-alcohol terminated oligomers at *m*/*z* 1246.8 of the PEA-Pro sample is reported in Figure 5.

As seen before, the most intense signals are due to ions originated by the cleavage of –O–CH_2_– bonds, because of a *β*-hydrogen-transfer rearrangement. However, this time, the end groups of the chain are different, leading to two different series’ of signals, Figure 5a, depending on the orientation of the 3-amino-1-propanol moiety (ions at *m*/*z* 225.10, 466.27, 707.44, 948.60, 1189.78 and their complementary at *m*/*z* 1044.75, 803.54, 562.38, 321.21, 80.03 and ions at *m*/*z* 264.16, 505.33, 746.49, 987.66, 1228.83 and their complementary at *m*/*z* 1005.68, 764.49, 523.33, 282.17). In the same way, the –CH_2_–CH_2_– bond cleavage in the sebacate moiety gives rise to two different series’ of ions, Figure 5b. *α,β*–unsaturated amide or ester, not discernible by MS, are originated as a function of the different orientation of the amino-alcohol (respectively, ions at *m*/*z* 95.0, 336.2, 577.3, 818.5, 1059.7 and their complementary at *m*/*z* 202.1, 449.3, 690.5, 931.6, 1172.8 and ions at *m*/*z* 151.1, 392.2, 633.4, 874.6, 1115.7 and their complementary at *m*/*z* 152.1, 393.2, 634.4, 875.6, 1116.7). Additionally, in this case, groups of ions differing by 14 *m*/*z* units are present (e.g., *m*/*z* 350.22, 364.24, 378.26 and 392.27), resulting from fragmentation of different –CH_2_–CH_2_– bonds in hydrocarbon chain of the sebacic moiety. Ions at *m*/*z* 280.17, 521.36, and 762.51, Figure 5c, can be assigned to product ions whose end groups can be formed by –O−CO− bond cleavages that lead to the formation of two ions: one bearing an aldehyde end group and the other with a HOCH=CH(CH_2_)_2_NHCO−/HCO(CH_2_)_3_NHCO− end group. Product ions bearing end groups originating from −CH_2_−CH_2_− (β−γ) bond cleavage, in the amino alcohol moiety, are also detected (e.g., *m*/*z* 251.12, 492.29 and 733.54). Several isobaric structures that differ by the position of a -CH_2_- group can be due to some of these ions. All the structures assigned are reported in Table S1.

**Figure 6:**
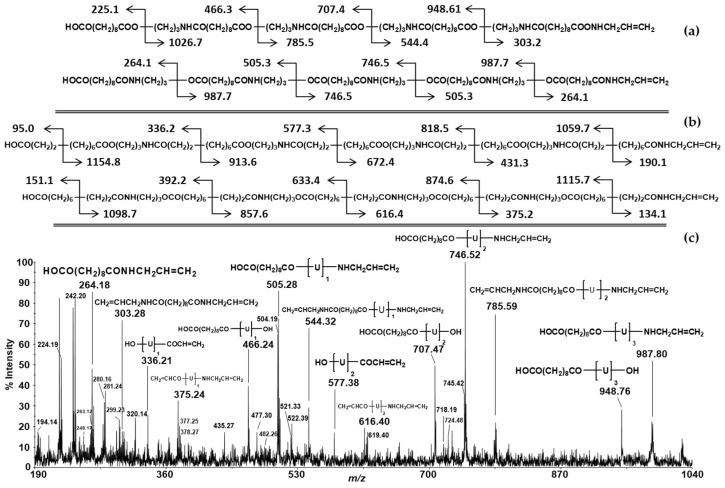
*m*/*z* values of the sodiated product ions originating from: (**a**) the *β*-hydrogen transfer and (**b**) the −CH_2_−CH_2_− (β−γ) bond cleavages in the sebacate moiety for the sodiated precursor ions at *m*/*z* 1228.8. (**c**) MALDI-TOF/TOF-MS/MS spectrum of the sodiated cyclic oligomers at *m*/*z* 1228.8 of the PEA-Pro sample (U= −NH(CH_2_)_3_OCO(CH_2_)_8_CO− or −O(CH_2_)_3_NHCO(CH_2_)_8_CO−; collision gas = argon).

Figure 6 displays the MALDI-TOF/TOF-MS/MS spectrum of the sodiated cyclic oligomers at *m*/*z* 1228.8 of the PEA-Pro sample. Cyclic oligomers may undergo ring opening at several backbone positions. However, since β-hydrogen-transfer is the main fragmentation mechanism, it is reasonable to assume that it is responsible for ring-opening reaction, resulting in linear chains terminated with carboxylic groups at one end and olefin group (CH_2_=CH(CH_2_)NHCO–) at the other end. Once again, different orientations of the 3-amino-1-propanol moiety led to two different series’ of ions for each fragmentation mechanism. In Figure 6, we report the *m*/*z* values of the product ions originating from: (a) β-hydrogen transfer and (b) –CH_2_–CH_2_– (β–γ) bond cleavages in the sebacate moiety.

**Figure 7:**
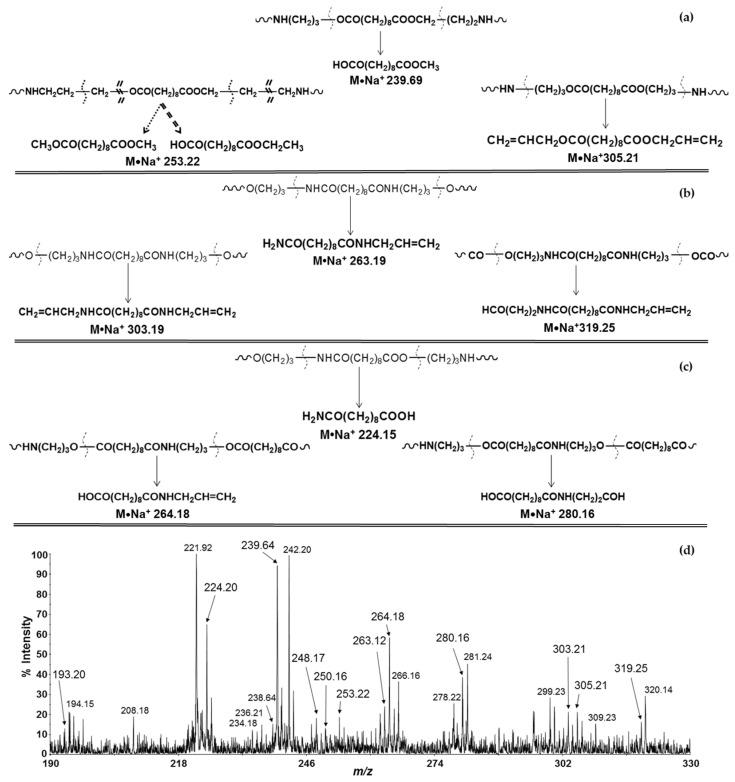
Bond cleavages leading to the diagnostic product ions from: (**a**) ester/ester, (**b**) amide/amide, and (**c**) ester/amide sequences. (**d**) Enlarged portion of MALDI-TOF/TOF-MS/MS spectrum of the sodiated cyclic oligomers at *m*/*z* 1228.8 of the PEA-Pro sample (collision gas = argon).

An enlarged portion of the same MALDI-TOF/TOF-MS/MS spectrum in the range *m*/*z* 190–330 is shown in Figure 7. Based on the structures of the identified ions, it is possible to exclude a regular alternate ester/amide sequence, i.e., infer that the orientation of 3-amino-1-propanol along the polymer chain is random. In fact, the ions at *m*/*z* 193, 239, 305 and 253 can be formed from ester/ester sequences (Figure 7a), and the structure of the ions at *m*/*z* 263, 319 and 303 can be originated by amide/amide sequences in the polymer chain (Figure 7b). Finally, the presence of the ions at *m*/*z* 224, 250, 264, 248 and 280 provides evidence for the existence of ester/amide sequences (Figure 7c). These ions arise from two bond cleavages of the polymer chains and the formation of some of them should involve high-energy processes.

**Figure 8:**
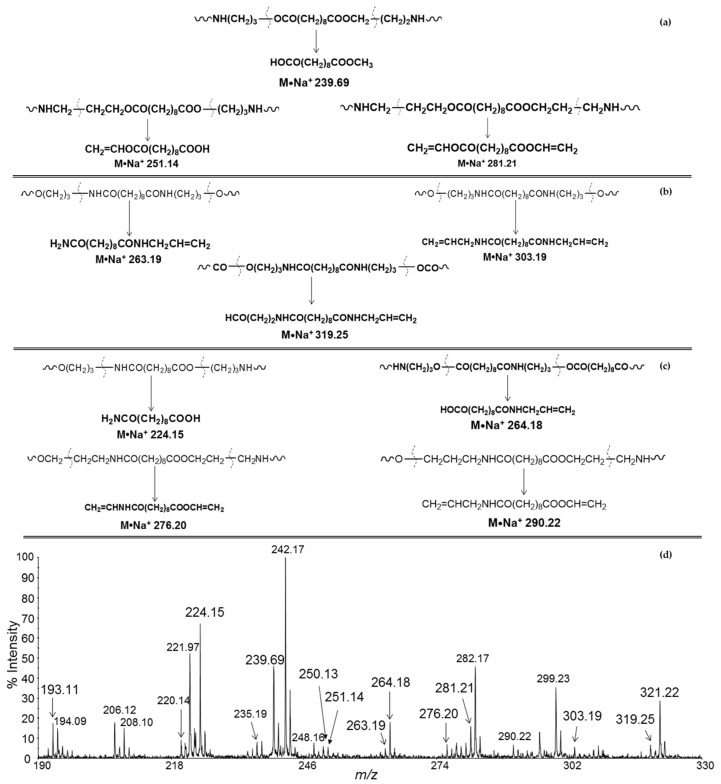
Bond cleavages leading to the diagnostic product ions from: (**a**) ester/ester, (**b**) amide/amide, and (**c**) ester/amide sequences. (**d**) Enlarged portion of MALDI-TOF/TOF-MS/MS spectrum of the sodiated dicarboxyl-terminated oligomers at *m*/*z* 1303.9 of the PEA-Pro sample (collision gas = argon).

In the same way, MALDI-TOF/TOF-MS/MS performed on the sodiated diamino alcohol terminated chains at *m*/*z* 1303.9 confirms that the ester and amide bonds are distributed randomly in the polymer chains. Figure 8 shows an enlarged portion of the MALDI-TOF/TOF-MS/MS spectrum in the range *m*/*z* 190–330. Additionally, in this case, it is possible identify sodiated product ions originated from: (a) ester/ester, (b) amide/amide and (c) ester/amide sequences.

### 3.4. Thermogravimetric Analysis (TGA)

**Figure 9:**
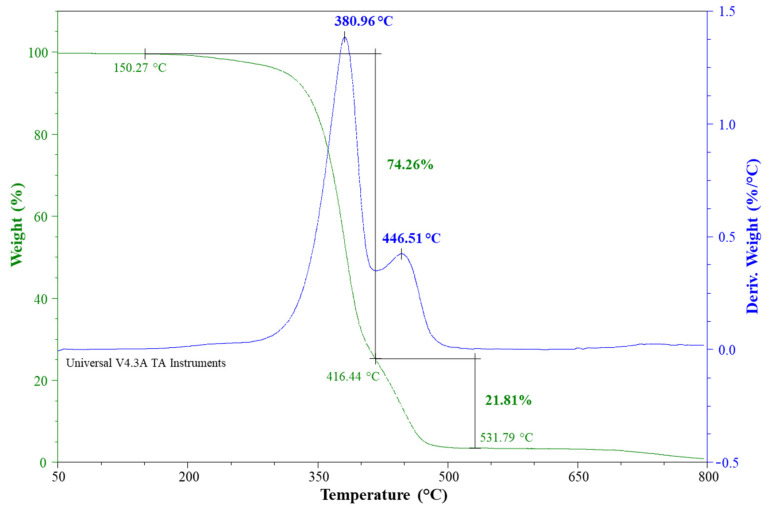
TGA (green trace) and DTG (blue trace) (heating rate 10 °C/min) for the PEA-Pro sample.

TGA provides much unique information that can be usefully combined with those obtained by other techniques, such as Py-GC/MS [18,22]. The thermal stability of PEA-Pro was evaluated by TGA at a heating rate of 10 °C/min under nitrogen atmosphere. In Figure 9, the weight loss (TGA, green trace) and its derivative (DTG, blue trace) as a function of temperature are presented together. Weight loss starts at about 150 °C, probably due to oligomers present in the sample and possibly to adsorbed water. The synthesis was performed at 180 °C [19] and this can justify the formation of degradation products, in agreement with the presence of ions detected in the mass spectra (Figure 3, species G and F) and of unassigned ions. Thermal degradation was found to happen in two main stages with respective maximum decomposition rates at around 380 °C and 446 °C. The first step (380 °C) corresponds to a weight loss of 74% and could be caused by the degradation of polyester segments; the second step (447 °C), with a mass loss of 22%, is related to the degradation of the amide segments [23]. Information about the degradation steps in TGA were used to optimize experimental conditions in Py-GC/MS analyses.

### 3.5. Pyrolysis-Gas Chromatography/Mass Spectrometry Analysis (Py-GC/MS)

The structure and thermal degradation of PEA-Pro was also investigated by pyrolysis coupled with GC/MS for the separation and detection/identification of the products of the pyrolysis. Two typical chromatograms of the pyrolysates of the PEA-Pro at 350 °C and 400 °C are shown in Figure S3a,b, respectively. At 450 °C, no signals were detected.

Possible compounds of the pyrolysis at 350 °C and 400 °C are listed in Table S2.

More degradation products were detected at 400 °C. Several vinyl-terminated compounds were identified, due to β-hydrogen scissions (occurring in β positions to both ester and amide bonds) and consequent rearrangements, and to dehydration processes. Additionally, carboxylic acids, alcohols, and as well as nitriles were assigned, in agreement with the literature [24]. At 350 °C, the main degradation product (RT = 22.049 min) originated from a β Hydrogen transfer mechanism or intramolecular cyclization accompanied by water loss. At higher degradation temperature (400 °C), the relative area of this signal (RT = 22.024 min) was lower, suggesting that the formation of those products is less favoured at higher temperatures. Overall, Py-GC/MS showed partial similarity between the degradation products and the main fragmentation mechanism detected by the MALDI tandem MS technique.

## 4. Conclusions

MALDI-TOF/TOF-MS/MS has proven to be a convenient technique for the structural characterization of a synthetic polyesteramide sample, providing detailed information on the fragmentation pathways and the ester/amide bond sequences in the polymer chains. The presence of different end groups does not influence the fragmentation of sodiated PEA-Pro oligomers, and similar series of product ions were observed in the MALDI-TOF/TOF-MS/MS spectra. Based on the structures of the product ions identified in the present work (Table S1), two main fragmentation cleavages have been proposed to occur most recurrently in PEA-Pro. In agreement with previous studies on similar polyesteramide samples, a β-hydrogen transfer rearrangement appears to be the most important fragmentation mechanism occurring in these MALDI-TOF/TOF-MS/MS experiments carried out with and without collision gas. The –CH_2_–CH_2_– (β–γ) bond cleavage in the sebacate moiety is reasonably driven by the formation of product ions bearing α,β-unsaturated ester or amide end groups. For each cleavage, two product ions are expected to appear in the MS/MS spectrum. However, the cleavage of some of the bonds yields only one ion. This should suggest that considerable differences exist in the sodium affinity of the product ions. In fact, when fragmentation occurs, the sodium cation can remain on one or the other of the two fragments, but the sodium affinity of the fragments most likely affects the distribution of the sodium cation between the species formed. MALDI-TOF/TOF-MS/MS analysis provided structural information concerning bond sequences in the polymer chains. The several new product ions present in MALDI-TOF/TOF-MS/MS spectra recorded using argon as the collision gas, above all in the low-mass range, proved diagnostic and made it possible to ascertain the presence of random sequences of ester and amide bonds in the polyesteramide sample.

Furthermore, TGA of PEA-Pro highlighted two main degradation steps at 380 °C (weight loss 74%) and 446 °C (weight loss 22%). Additionally, structural characterization performed by Py-GC/MS provided a partial similarity between the degradation products and the main fragmentation mechanism detected by the MALDI technique.

NMR analysis showed the presence of hydroxyl and amino terminal groups as well carboxylic groups of the sebacate moiety. As a plus, NMR showed that the 3-amino-1-propanol termination may present a free hydroxyl or amine group but, from a quantitative point of view, that hydroxyl and carboxyl termination have the same abundance, while the amine termination is 2.7-times less frequent.

## Data Availability

The data presented in this study are available on request from the corresponding author.

## Supplementary Materials

The following supporting information can be downloaded at: https://www.mdpi.com/article/10.3390/polym14081500/s1, Figure S1: Partial g-COSY spectrum of PEA-Pro in 6d-DMSO at 50 °C showing the 4.2–1.0 ppm range. Figure S2: Partial g-COSY spectrum of PEA-Pro in 6d-DMSO at 50 °C showing the 8.0–2.6 ppm range. Figure S3: Total ion current (TIC) trace of pyrolysis products of PEA-Pro at (**a**) 350 °C and (**b**) 400 °C; Table S1: Structure assignments of product ions appearing in the MALDI-TOF/TOF-MS/MS spectra of the PEA-Pro sample (precursor ions at *m*/*z* 1189.8, 1228.8, 1246.8 and 1303.9). Table S2: Structure assignment of the degradation products derived from the pyrolysis of the PEA-pro at 350 and 400 °C.
