# Supplementary material for: Sequencing Biodegradable and Potentially Biobased Polyesteramide of Sebacic Acid and 3-Amino-1-propanol by MALDI TOF-TOF Tandem Mass Spectrometry
Source: Polymers (Basel). 2022 Apr 7;14(8):1500. doi: 10.3390/polym14081500 (PMC9032766; doi:10.3390/polym14081500)
Supplement: Supplementary file 1 [file polymers-14-01500-s001.zip › polymers-1660284-supplementary.pdf]

**Supplemental Information:**

# Sequencing Biodegradable and Potentially Biobased Polyesteramide of Sebacic Acid and 3-Amino-1-Propanol by MALDI TOF-TOF Tandem Mass Spectrometry

**Paola Rizzarelli [1,*], Stefania La Carta [2], Emanuele Francesco Mirabella [1], Marco Rapisarda [1] and Giuseppe Impallomeni [1]**

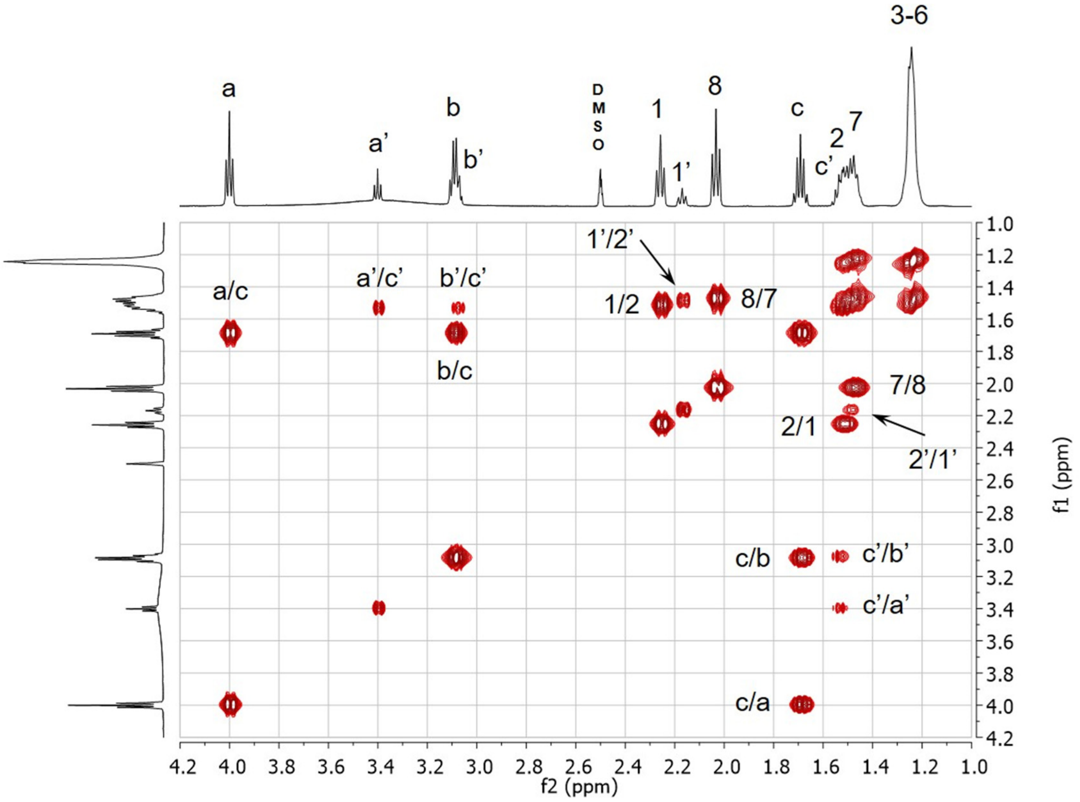


**Figure S1.** Partial g-COSY spectrum of PEA-Pro in 6d-DMSO at 50 °C showing the 4.2 – 1.0 ppm range.

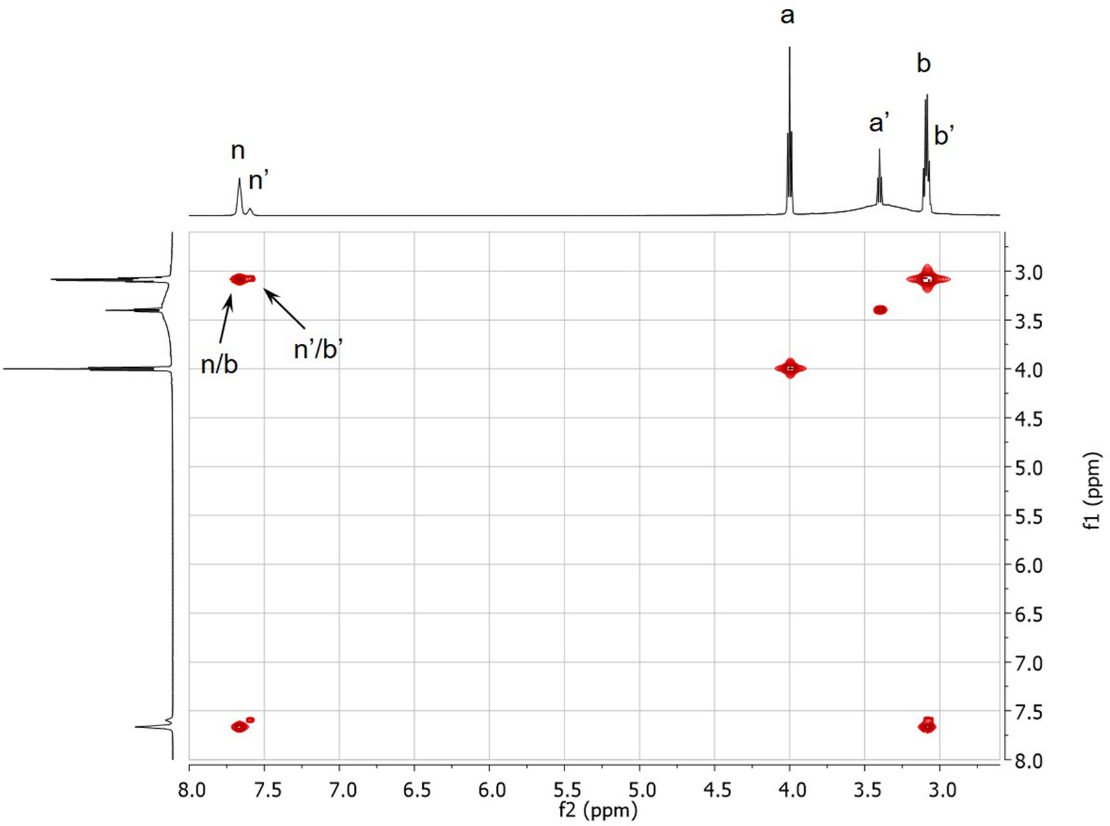


**Figure S2.** Partial g-COSY spectrum of PEA-Pro in 6d-DMSO at 50°C showing the 8.0 – 2.6 ppm range.

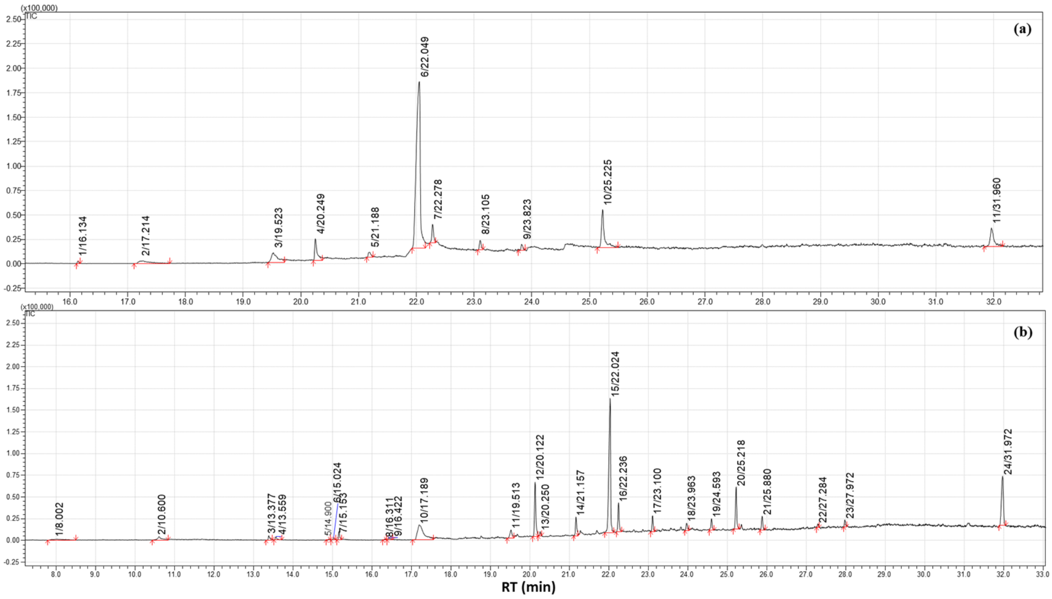


**Figure S3.** Total ion current (TIC) trace of pyrolysis products of PEA-Pro at **(a)** 350 °C and **(b)** 400 °C.

**Table S1.** Structure assignments of product ions appearing in the MALDI-TOF/TOF-MS/MS spectra of the PEA-Pro sample (precursor ions at *m*/*z* 1189.8, 1228.8, 1246.8 and 1303.9).

| Structure | n | [M+Na+] | [M+H+] |
|---|---|---|---|
| | 4 | 1189.78 | |
| | 3 | 948.61 | |
| $HO{-}[U]_n{-}CO(CH_2)_8COOH$ | 2 | 707.44 | / |
| | 1 | 466.27 | |
| | 0 | 225.10 | |
| | 4 | 1228.83 | |
| | 3 | 987.66 | |
| $H_2C{=}CHCH_2NH{-}[U]_n{-}CO(CH_2)_8COOH$ | 2 | 746.48 | / |
| | 1 | 505.32 | |
| | 0 | 264.18 | |
| | 5 | 1228.83 | |
| 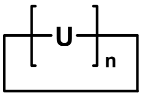 | 4 | 987.66 | |
| | 3 | 746.48 | |
| | 2 | 505.32 | |
| | 1 | 264.18 | 242.18 |
| $CH_2{=}CHCO{-}[U]_n{-}OH$ | 4 | 1059.83 | |
| | 3 | 818.50 | |
| | 2 | 577.37 | / |
| $HCO{-}[U]_n{-}OCH{=}CH_2$ | 1 | 336.21 | |
| | 0 | 95.04 | |
| $CH_2{=}CHCH_2CO{-}[U]_n{-}OH$ | 3 | 832.52 | |
| $HCO{-}[U]_n{-}OCH_2CH{=}CH_2$ | 2 | 591.35 | / |
| | 1 | 350.18 | |
| $CH_3CO{-}[U]_n{-}OCH{=}CH_2$ | 0 | 109.01 | |
| $CH_2{=}CH(CH_2)_2CO{-}[U]_n{-}OH$ | 3 | 846.82 | |
| | 2 | 605.63 | / |
| $CH_2{=}CHCO{-}[U]_n{-}OCH_2CH_3$ | 1 | 364.34 | |
| | 0 | 123.09 | |
| | 4 | 1044.63 | 1022.62 |
| | 3 | 803.52 | 781.48 |
| $CH_2{=}CHCH_2NH{-}[U]_n{-}H$ | 2 | 562.38 | 540.40 |
| | 1 | 321.22 | 299.23 |
| | 0 | 80.59 | 58.04 |
| | 2 | 744.47 | |
| $CH_2{=}CH_2CO{-}[U]_n{-}O(CH_2)_3NHCO(CH_2)_4CH$ | 1 | 503.30 | / |
| | 0 | 262.13 | |
| $HOCO(CH_2)_8CO{-}[U]_n{-}NH_2$ | 1 | 465.30 | / |
| | 0 | 224.15 | |
| | 3 | 1005.68 | |
| $HO{-}[U]_n{-}H$ | 2 | 764.50 | / |
| | 1 | 523.34 | |
| | 0 | 282.17 | |
| | 4 | 1172.80 | |
| | 3 | 931.74 | |
| $CH_2{=}CH(CH_2)_4CO{-}[U]_n{-}O(CH_2)_3NH_2$ | 2 | 690.53 | / |
| | 1 | 449.33 | |
| | 0 | 208.10 | |
| $CH_2{=}CH(CH_2)_3CO{-}[U]_n{-}O(CH_2)_3NH_2$ | 3 | 917.60 | |
| $CH_3(CH_2)_4CO{-}[U]_n{-}NH(CH_2)_2CHO$ | 2 | 676.49 | / |
| | 1 | 435.32 | |
| $CH_2{=}CHCH_2CO{-}[U]_n{-}O(CH_2)_3NHCHO$ | 0 | 194.09 | |

| Structure | n | [M+Na$^+$] | [M+H$^+$] |
|---|---|---|---|
| $CH_2{=}CHCO{-}[U]_n{-}O(CH_2)_3NHCOCH_3$ | | | |
| $CH_2{=}CH(CH_2)_2CO{-}[U]_n{-}O(CH_2)_3NH_2$ | 3 | 903.59 | / |
| | 2 | 662.42 | |
| | 1 | 421.30 | |
| | 0 | 180.10 | |
| $CH_2{=}CHCH_2CO{-}[U]_n{-}O(CH_2)_3NH_2$ | 3 | 889.74 | / |
| | 2 | 648.40 | |
| $CH_3(CH_2)_2CO{-}[U]_n{-}NH(CH_2)CHO$ | 1 | 407.29 | |
| | 0 | 166.09 | |
| $CH_2{=}CHCO{-}[U]_n{-}O(CH_2)_3NH_2$ | 4 | 1116.73 | / |
| | 3 | 875.61 | |
| | 2 | 634.49 | |
| $CH_3(CH_2)_5CO{-}[U]_n{-}NH_2$ | 1 | 393.28 | |
| | 0 | 152.06 | |
| $HOCO(CH_2)_8CO{-}[U]_n{-}NH(CH_2)_2CHO$ | 3 | 1003.76 | / |
| | 2 | 762.51 | |
| $CH_3CH_2O{-}[U]_n{-}CO(CH_2)_8CONHCH_2CH_3$ | 1 | 521.36 | |
| | 0 | 280.17 | |
| $CH_2{=}CHCH_2NH{-}[U]_n{-}CO(CH_2)_8CONHC$ | 3 | 1026.91 | / |
| | 2 | 785.69 | |
| | 1 | 544.36 | |
| | 0 | 303.19 | |
| $CH_2{=}CH(CH_2)_4CO{-}[U]_n{-}NHCH_2CH{=}CH_2$ | 3 | 913.62 | / |
| | 2 | 672.45 | |
| $CH_2{=}CH(CH_2)_5CO{-}[U]_n{-}NHCH{=}CH_2$ | 1 | 431.28 | |
| | 0 | 190.11 | |
| $CH_2{=}CHCH_2CO{-}[U]_n{-}NHCH{=}CH_2$ | 4 | 1089.80 | / |
| | 3 | 857.68 | |
| $CH_2{=}CHCO{-}[U]_n{-}NHCH_2CH{=}CH_2$ | 2 | 616.40 | |
| | 1 | 375.27 | |
| | 0 | 134.08 | |
| $H{-}[U]_n{-}NH(CH_2)CHO$ | 4 | 1060.79 | / |
| | 3 | 819.59 | |
| | 2 | 578.44 | |
| | 1 | 337.25 | |
| | 0 | 96.47 | |
| $CH_2{=}CH(CH_2)_6CO{-}[U]_n{-}NH_2$ | | | |
| $CH_3(CH_2)_5CO{-}[U]_n{-}NHCH{=}CH_2$ | 2 | 660.54 | / |
| | 1 | 419.30 | |
| $CH_3(CH_2)_4CO{-}[U]_n{-}NHCH_2CH{=}CH_2$ | 0 | 178.11 | |
| $CH_2{=}CH(CH_2)_2CO{-}[U]_n{-}NH(CH_2)CHO$ | | | |
| $CH_2{=}CH(CH_2)_4CO{-}[U]_n{-}NH_2$ | | | |
| $CH_2{=}CHCO{-}[U]_n{-}NH(CH_2)_2CHO$ | 2 | 632.42 | / |
| | 1 | 391.25 | |
| $CH_3(CH_2)_2CO{-}[U]_n{-}NHCH_2CH{=}CH_2$ | 0 | 150.08 | |
| $CH_3(CH_2)_3CO{-}[U]_n{-}NHCH{=}CH_2$ | | | |
| $CH_2{=}CH(CH_2)_2CO{-}[U]_n{-}NH_2$ | 2 | 604.39 | / |
| | 1 | 363.22 | |
| | 0 | 122.05 | |

| Structure | n | [M+Na$^+$] | [M+H$^+$] |
|---|---|---|---|
| $CH_3CO-[U]_n-NHCH_2CH=CH_2$<br>$CH_2=CHCH_2CO-[U]_n-NH_2$<br>$CH_3CO-[U]_n-NHCH=CH_2$<br>$HCO-[U]_n-NHCH_2CH=CH_2$ | 2<br>1<br>0 | 590.37<br>349.20<br>108.03 | / |
| $HCO(CH_2)_8CO-[U]_n-NHCH_2CH=CH_2$ | 2<br>1<br>0 | 730.54<br>489.38<br>248.16 | / |
| $H_2NCO(CH_2)_8CO-[U]_n-NHCH_2CH=CH_2$ | 2<br>1<br>0 | 745.63<br>504.33<br>263.19 | / |
| $HOCO(CH_2)_8CO-[U]_n-NH(CH_2)_2CH_3$ | 3<br>2<br>1<br>0 | 989.68<br>748.51<br>507.29<br>266.16 | / |
| $NH_2CO(CH_2)_8CO-[U]_n-O(CH_2)_3NH_2$<br>$CH_3CH_2O-[U]_n-CO(CH_2)_8COOCH_2CH_3$ | 3<br>2<br>1<br>0 | 1004.78<br>763.58<br>522.39<br>281.25 | / |
| $CH_2=CH(CH_2)_6CO-[U]_n-OH$<br>$CH_2=CH(CH_2)_4CO-[U]_n-OCH_2CH_3$<br>$CH_3(CH_2)_4CO-[U]_n-OCH_2CH=CH_2$ | 3<br>2<br>1<br>0 | 902.57<br>661.49<br>420.45<br>179.19 | / |
| $CH_2=CH(CH_2)_5CO-[U]_n-OH$<br>$CH_3(CH_2)_3CO-[U]_n-OCH_2CH=CH_2$<br>$CH_3(CH_2)_4CO-[U]_n-OCH=CH_2$ | 2<br>1<br>0 | 647.42<br>406.25<br>165.11 | / |
| $CH_2=CH(CH_2)_3CO-[U]_n-NH_2$ | 2<br>1<br>0 | 618.49<br>377.23<br>136.06 | / |
| $CH_2=CH(CH_2)_4CO-[U]_n-OH$<br>$CH_2=CH(CH_2)_2CO-[U]_n-OCH_2CH_3$<br>$CH_3(CH_2)_3CO-[U]_n-OCH=CH_2$<br>$CH_3(CH_2)_2CO-[U]_n-OCH_2CH=CH_2$ | 4<br>3<br>2<br>1<br>0 | 1115.74<br>874.57<br>633.40<br>392.23<br>151.06 | / |
| $CH_2=CH(CH_2)_3CO-[U]_n-OH$<br>$CH_2=CHCH_2CO-[U]_n-OCH_2CH_3$<br>$CH_3(CH_2)_2CO-[U]_n-OCH=CH_2$ | 2<br>1<br>0 | 619.49<br>378.26<br>137.07 | / |

| Structure | n | [M+Na+] | [M+H+] |
|---|---|---|---|
| $CH_3(CH_2)_4CO-[U]_n-OH$ | 1<br>0 | 380.23<br>139.06 | / |
| $CH_2=CH(CH_2)_5CO-[U]_n-NH_2$<br>$CH_3(CH_2)_3CO-[U]_n-NHCH_2CH=C$<br>$CH_2=CHCH_2CO-[U]_n-NH(CH_2)_2CHO$ | 3<br>2<br>1<br>0 | 887.65<br>646.48<br>405.27<br>164.10 | / |
| $CH_3(CH_2)_4CO-[U]_n-NH_2$ | 2<br>1<br>0 | 620.40<br>379.25<br>138.08 | / |
| $CH_3(CH_2)_5CO-[U]_n-OCH_2CH_3$ | 2<br>1<br>0 | 663.44<br>422.33<br>181.07 | / |
| $CH_2=CH(CH_2)_4CO-[U]_n-NH(CH_2)_2CHO$<br>$CH_2=CHCO-[U]_n-O(CH_2)_3NHCOCH=$ | 2<br>1<br>0 | 688.52<br>447.31<br>206.12 | / |
| $CH_2=CHCH_2CO-[U]_n-O(CH_2)_3NHCO$<br>$CH_2=CH(CH_2)_5CO-[U]_n-NH(CH_2)_2CHC$<br>$CH_2=CH(CH_2)_3CO-[U]_n-O(CH_2)_3NHCH$ | 2<br>1<br>0 | 702.45<br>461.41<br>220.13 | / |
| $CH_3(CH_2)_5CO-[U]_n-NH(CH_2)_2CHO$<br>$CH_2=CH(CH_2)_2CO-[U]_n-O(CH_2)_3NHCO$<br>$CH_2=CHCO-[U]_n-O(CH_2)_3NHCO(CH_2$<br>$CH_2=CH(CH_2)_4CO-[U]_n-O(CH_2)_3NHC$ | 3<br>2<br>1<br>0 | 945.73<br>704.53<br>463.35<br>222.06 | / |
| $CH_2=CH(CH_2)_3CO-[U]_n-O(CH_2)_3NH$<br>$CH_2=CHCH_2CO-[U]_n-O(CH_2)_3NHC$<br>$CH_2=CHCONH(CH_2)_3O-[U]_n-CO(CH_2)_3$ | 3<br>2<br>1<br>0 | 959.69<br>718.52<br>477.33<br>236.14 | / |
| $HOCO(CH)_8CO-[U]_n-OCH=CH_2$ | 2<br>1<br>0 | 733.54<br>492.29<br>251.12 | / |
| $CH_2=CH(CH_2)_4CO-[U]_n-O(CH_2)_3NHC$<br>$CH_2=CH(CH_2)_2CO-[U]_n-O(CH_2)_3NHC$<br>$CH_2=CHCH_2CO-[U]_n-O(CH_2)_3NHCO(CH$<br>$CH_2=CHCO-[U]_n-O(CH_2)_3NHCO(CH_2$<br>$CH_2=CHO-[U]_n-CO(CH_2)_8CONH_2$ | 3<br>2<br>1<br>0 | 973.17<br>732.57<br>491.34<br>250.14 | / |

| Structure | n | [M+Na+] | [M+H+] |
|---|---|---|---|
| $HOCO(CH)_8CO-[U]_n-NHCH=CH_2$ | | | |
| $CH_2=CH(CH_2)_6CO-[U]_n-O(CH_2)_3NHCO$ | 2<br>1<br>0 | 772.71<br>531.44<br>290.22 | / |
| $CH_2=CH(CH_2)_3CO-[U]_n-O(CH_2)_3NH($ | 3<br>2<br>1<br>0 | 1029.69<br>788.52<br>547.35<br>306.18 | / |
| $CH_3(CH_2)_5CO-[U]_n-O(CH_2)_3NHCO(CH$<br>$HOCH=CHCH_2NH-[U]_n-CO(CH_2)_8CO$<br>$HO(CH_2)_3NH-[U]_n-CO(CH_2)_8COOCH=$ | 3<br>2<br>1<br>0 | 1031.11<br>790.67<br>549.38<br>308.21 | / |
| $CH_2=CHCO-[U]_n-H$ | 3<br>2<br>1<br>0 | 802.51<br>561.34<br>320.17<br>79.00 | / |
| $CH_2=CH(CH_2)_6CO-[U]_n-OCH_2CH_3$ | 3<br>2<br>1<br>0 | 930.63<br>689.46<br>448.31<br>207.14 | / |

* U = $-NH(CH_2)_3OCO(CH_2)_8CO-$ or $-O(CH_2)_3NHCO(CH_2)_8CO-$.

**Table S2.** Structure assignment of the degradation products derived from the pyrolysis of the PEA-pro at 350 and 400 °C.

| RT (min) | Possible structure (Mol Wt) | Ion EI[a] (*m/z*) | A%[b] 350 °C | A%[b] 400 °C |
|---|---|---|---|---|
| 8.002 | **$CH_3(CH_2)_4COOH$* (116)** | 73 | / | 1.31 |
| 10.600 | **$CH_3(CH_2)_7COOH$* (158)** | 129 | / | 1.99 |
| 13.377 | **$CH_3(CH_2)_4CONHCH_2CH=CH_2$* (155)** | 140 | / | 0.96 |
| 13.559 | **$CH_3(CH_2)_5CONHCH_2CH=CH_2$* (169)** | 126 | / | 0.63 |
| 14.900 | **unidentified** | 99 | / | 0.07 |
| 15.024 | **$CH_3(CH_2)_6CONHCH_2CH=CH_2$ (183)** | 112 | / | 0.43 |
| 15.154 | **unidentified** | 168 | / | 1.33 |
| 16.134 | **unidentified** | 177 | 0.28 | / |
| 16.311 | **unidentified** | 112 | / | 0.15 |
| 16.422 | **unidentified** | 112 | / | 0.45 |
| 17.214/ 17.189 | **$HOCO(CH_2)_8COOH$ (202)** | 138 | 2.96 | 12.15 |
| 19.522/ 19.513 | **$NC(CH_2)_8CN$ (164)** | 164 | 5.92 | 2.54 |
| 20.122 | **$NC(CH_2)_8CONHCH_2CH=CH_2$ (222)** | 221 | / | 9.69 |
| 20.249 | **$CH_3(CH_2)_6CONHCH_2CH=CH_2$ (183)** | 183 | 5.30 | 0.55 |
| 21.188/ 21.158 | **$NC(CH_2)_8CONHCH=CH_2$ (208)** | 207 | 1.28 | 3.43 |

| RT (min) | Possible structure (Mol Wt) | Ion EI[a] (*m*/*z*) | A%[b] 350 °C | A%[b] 400 °C |
|---|---|---|---|---|
| 22.049/ 22.024 | 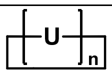 **or $HOCO(CH_2)_8CONHCH_2CH{=}CH_2$ (241)** | 241 | 61.83 | 30.49 |
| 22.278/ 22.236 | 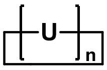 **or $HOCO(CH_2)_8CONHCH_2CH{=}CH_2$ (241)** | 241 | 3.22 | 4.76 |
| 23.100 | **$CH_2{=}CHCH_2OCO(CH_2)_8CONHCH_2CH{=}CH_2$ (281)** | 281 | / | 2.43 |
| 23.104 | **unidentified** | 182 | 1.52 | / |
| 23.822 | **$CH_3CH_2OCO(CH_2)_8CONHCH_2CH{=}CH_2$ (269)** | 269 | 0.91 | / |
| 23.963 | **unidentified** | 281 | / | 1.61 |
| 24.593 | **$CH_2{=}CHCH_2NHCO(CH_2)_8CONH(CH_2)_3OH$ (298)** | 281 | / | 2.03 |
| 25.225/ 25.218 | **$CH_3(CH_2)_2NH(CH_2)_3OCO(CH_2)_8CN$ (282) or $CH_2{=}CHCH_2OCO(CH_2)_8CONHCH_2CH{=}CH_2$ or $CH_2{=}CHCH_2COO(CH_2)_3NHCO(CH_2)_6CH{=}CH_2$ (281)** | 265 | 10.47 | 6.73 |
| 25.880 | **$CH_3(CH_2)_2O(CH_2)_3NHCO(CH_2)_8CN$ (282)** | 281 | / | 2.36 |
| 27.284 | **unidentified** | 429 | / | 0.59 |
| 27.972 | **unidentified** | 429 | / | 1.05 |
| 31.960 | **unidentified** | 429 | 6.31 | / |
| 31.972 | **unidentified** | 429 | / | 12.27 |

a: Molecular ion or major fragment; b: Area %.
